# Liver-Specific Expression of Transcriptionally Active SREBP-1c Is Associated with Fatty Liver and Increased Visceral Fat Mass

**DOI:** 10.1371/journal.pone.0031812

**Published:** 2012-02-21

**Authors:** Birgit Knebel, Jutta Haas, Sonja Hartwig, Sylvia Jacob, Cornelia Köllmer, Ulrike Nitzgen, Dirk Muller–Wieland, Jorg Kotzka

**Affiliations:** 1 Institute of Clinical Biochemistry and Pathobiochemistry, German Diabetes Center at the Heinrich-Heine-University Duesseldorf, Leibniz Center for Diabetes Research, Duesseldorf, Germany; 2 Institute for Diabetes Research, Department of General Internal Medicine, Asklepios Clinic St. Georg, Medical Faculty of the Semmelweis University, Hamburg, Germany; South Texas Veterans Health Care System and University Health Science Center San Antonio, United States of America

## Abstract

The pathogenesis of fatty liver is not understood in detail, but lipid overflow as well as *de novo* lipogenesis (DNL) seem to be the key points of hepatocyte accumulation of lipids. One key transcription factor in DNL is sterol regulatory element-binding protein (SREBP)-1c. We generated mice with liver-specific over-expression of mature human SREBP-1c under control of the albumin promoter and a liver-specific enhancer (alb-SREBP-1c) to analyze systemic perturbations caused by this distinct alteration. SREBP-1c targets specific genes and causes key enzymes in DNL and lipid metabolism to be up-regulated. The alb-SREBP-1c mice developed hepatic lipid accumulation featuring a fatty liver by the age of 24 weeks under normocaloric nutrition. On a molecular level, clinical parameters and lipid-profiles varied according to the fatty liver phenotype. The desaturation index was increased compared to wild type mice. In liver, fatty acids (FA) were increased by 50% (p<0.01) and lipid composition was shifted to mono unsaturated FA, whereas lipid profile in adipose tissue or serum was not altered. Serum analyses revealed a ∼2-fold (p<0.01) increase in triglycerides and free fatty acids, and a ∼3-fold (p<0.01) increase in insulin levels, indicating insulin resistance; however, no significant cytokine profile alterations have been determined. Interestingly and unexpectedly, mice also developed adipositas with considerably increased visceral adipose tissue, although calorie intake was not different compared to control mice. In conclusion, the alb-SREBP-1c mouse model allowed the elucidation of the systemic impact of SREBP-1c as a central regulator of lipid metabolism *in vivo* and also demonstrated that the liver is a more active player in metabolic diseases such as visceral obesity and insulin resistance.

## Introduction

Liver lipid content is influenced by serious, common metabolic diseases such as visceral obesity, lipid disorders or type 2 diabetes. This lipid overload has destructive effects on cell functionality and viability, as initially pronounced by Unger in the “lipoptoxicity hypothesis” [1]. The mechanisms of ectopic hepatic lipid accumulation in the development of fatty liver are still under debate, but the interaction of increased lipolysis, lipid transport or hepatic *de novo* lipogenesis (DNL) is definitely involved [2], [3]. One possible approach to narrow down the impact of each distinct component of the three-part model of hepatic lipid accumulation is to intervene at one specific branch and to analyze the physiological and phenotypical outcome in detail.

Hepatic lipids originate mainly from the release of stored lipids from adipocytes, which also occurrs in the case of insulin resistance. This process is thought to account for the majority of lipid accumulated in hepatocytes. On the other hand, the direct induction of hepatic DNL synthesis, i.e. the production of fatty acids from carbohydrates or amino acids, might be the initial step in the cycle of lipid accumulation in hepatocytes [4].

DNL describes the excess flow of glucose from carbohydrate stores into the hepatic acetyl-CoA pool via glycolysis for the production of triglycerides. Any surplus of carbohydrates that is not directly oxidized for energy is metabolized to triglycerides for storage. Generally, DNL is modified by total energy intake, dietary fat/carbohydrate ratio, or glucose and/or insulin concentration [5]–[15]. In healthy individuals, the portion of DNL in hepatic lipid content seems to be approximately 5% of total lipids, but in patients with non-alcoholic steatohepatitis (NASH), this portion can increase to 26% [2].

DNL can be triggered by multiple mechanisms, including increased expression of lipogenic enzymes by several specific transcription factors; this is particularly true for members of the SREBP family. One of them, i.e. SREBP-1c, controls hepatic DNL primarily by regulation of expression of genes involved in DNL, lipid homeostasis and glucose metabolism [16]–[21]. Accordingly, hepatic expression of SREBP-1c and its target genes has been shown to be increased in human fatty liver compared to healthy individuals [22]–[26].

SREBP-1c belongs to the basic-helix-loop-helix transcription factors. The SREBP family consists of the isoforms SREBP-1 and SREBP-2 that are encoded by two different genes [27], [28]. In contrast to SREBF-2, SREBF-1 is transcribed into two splice variants: SREBP-1a and SREBP-1c [29]. SREBP-2 predominantly regulates cholesterol synthesis; the isoform SREBP-1a controls cholesterol and lipid synthesis, whereas SREBP-1c solely regulates the synthesis of fatty acids [17], [22], [30]–[32].

SREBP-1c is transcribed and translated into precursor molecules. These are transcriptionally inactive and are embedded in the membrane of the endoplasmatic reticulum. The release of the transcriptionally active domain by sequential two-step proteolytic machinery is controlled by complex metabolic regulation [31], [33].

The mature forms of SREBPs translocate into the nucleus and facilitate gene expression of target genes. SREBP-1c has been shown to be key mediator in induction of DNL, independent of food composition, and is a convergence point of hormones, growth factors and inflammatory cytokines in the regulation of genes involved in lipid metabolism [22], [34]–[36]. Therefore, this transcription factor is a favored candidate to analyse the role of hepatic DNL in the the develpoment of fatty liver disease.

To elucidate the role of increased *de novo* lipid synthesis in the pathogenesis of hepatic lipid accumulation, we generated a novel mouse model that overexpresses the transcriptionally active domain of human SREBP-1c under control of the liver-pecific albumin promoter. In this report, we focus on the description of the alb-SREBP-1c mouse model and show that the expression of genes playing a central role in lipid metabolism as well as total fatty acid composition are altered in the livers of these mice. Correspondingly, hepatic lipid accumulation occurred featuring a fatty liver phenotype. Moreover, increased visceral adipose tissue and an altered adipose tissue distribution were found, although caloric intake was unaltered amongst control mice.

## Results

### Increased hepatic lipid accumulation, gene expression and *de novo* lipogenesis caused by liver-specific over-expression of the transcriptionally active N-terminal domain of human SREBP-1c

**Figure 1:**
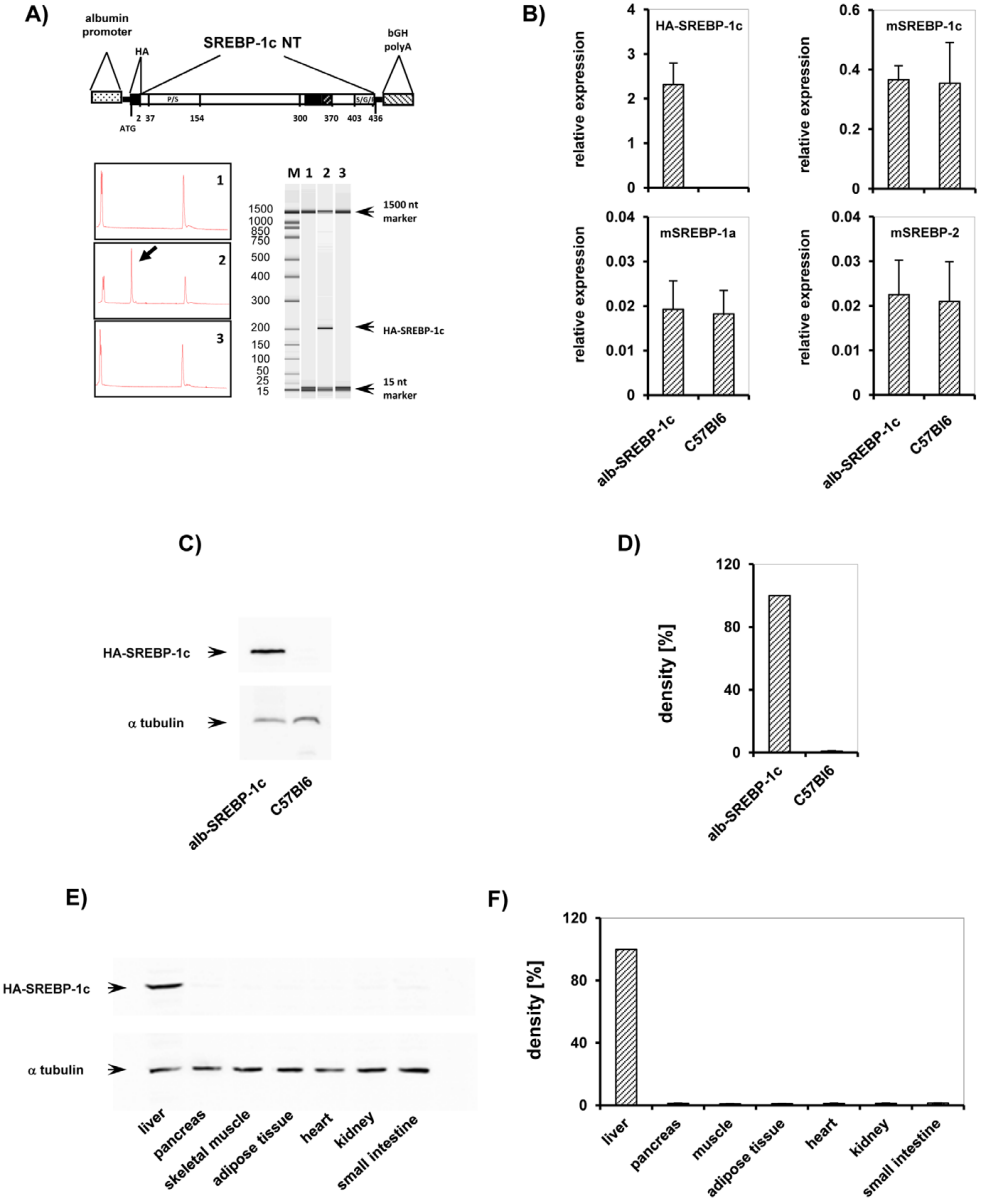
Tissue-specific over-expression of alb-SREBP-1c *in vivo*. (A) Scheme of the DNA constructs used to generate transgenic mice. The transcriptionally active N-terminal domain of the human SREBP-1c gene (aa 1–436 including a 5′-HA-tag (YPYDVPDYA) was inserted into a vector construct containing the mouse albumin promoter, a liver-specific enhancer element and a polyadenylation site. The SREBP-1c expression cassette was released by BssHI restriction for microinjection into male pronuclei of zygotes derived from C57Bl6 mice. (A) Verification of transgene insertion into genomic DNA was performed by PCR. M: size marker, genomic DNA of lane 1: C57Bl6, 2: alb-SREBP-1c, and lane 3: no template control. (B) Validation of transgene expression of alb-SREBP-1c animal model on mRNA level by RT-PCR. RNA extracted from snap-frozen liver biopsies from male alb-SREBP-1c and C57Bl6 mice was analyzed by RT-PCR with transgene human SREBP-1c (HA-SREBP-1c) , mouse SREBP-1a (m-SREBP-1a), mouse SREBP-1c (m-SREBP-1c) and mouse SREBP-2 (m-SREBP-2) specific primers and probe. The relative RNA amount shown in arbitrary units was calculated and plotted ± S.D. Graphs represent data from four male mice per genotype, each analyzed in triplicate (p<0.01). (C) Verification of transgene expression on protein level in liver. Protein extracts of snap-frozen liver biopsies from lane 1: alb-SREBP-1c, 2: C57Bl6 mice were separated by SDS-PAGE and blotted on nitrocellulose membrane. The membrane was probed with HA-specific antibody to determine the HA-tag of the transgene construct. A representative experiment is shown. For normalizing, blots were probed with α-tubulin antibody. (D) Graphs show densitometry evaluation of n = 5 independent experiments. (E) Tissue-specific expression of alb-SREBP-1c. Protein extracts of lane 1: liver, 2: pancreas, 3: skeletal muscle, 4: adipose tissue, 5: heart, 6: kidney and 7: small intestine were separated by SDS-PAGE, blotted and probed with HA-specific antibody. A representative experiment is shown. The arrow indicates HA-tagged SREBP-1c. For normalizing, blots were probed with α-tubulin antibody. (F) Graphs show densitometry evaluation of n = 5 independent experiments.

The role of SREBP-1c in hepatic *de novo* lipogenesis (DNL) has been shown in *in vitro* analyses and cellular models [16]–[21]. To study the role of DNL in lipid accumulation in liver, we generated mice that overexpress the transcriptionally active domain of human SREBP-1c specificly in liver. To restrict expression of the transgene solely to the liver and to enable a permanent high level of expression throughout development, we decided to host the transgene under the control of the albumin promoter as well as a liver-specific enhancer, and we designated them as ‘alb-SREBP-1c’ (Figure 1A).

The presence of the transgene was monitored by genotyping of the intersection of albumin-promoter and coding sequence of human SREBP-1c (Figure 1A). At mRNA level, the expression of the transgene in liver could only be monitored in the alb-SREBP-1c animals, whereas the expression of endogenous SREBP-1c, the isoform SREBP-1a, and SREBP-2 were unaltered between transgenes and control mice (Figure 1B). At protein level, the transgene could be specifically detected by HA-tag antibody to discriminate it from the endogenous SREBP-1. This signal was only detected in the livers of alb-SREBP-1c mice but not in C57Bl6 control animals (Figure 1C, 1D). Protein expression studies showed that the construct was strictly restricted to the liver in alb-SREBP-1c animals (Figure 1E, 1F).

The gene expression levels of a panel of key metabolic enzymes targeted by SREBP-1c were investigated in liver in order to monitor the effect of over-expression of the human transcription factor on gene regulation in mouse liver (Table 1).

**Table 1:** Gene expression of metabolic genes in liver.

	C57Bl6	alb-SREBP-1c
**FAS**	8.62±4.61	106.34±51.24^**^
**SCD1**	373.87±159.52	2017.10±159.37^**^
**FADS1**	45.54±8.18	119.12±27.88^**^
**FADS2**	20.98±7.09	82.78±18.89^**^
**EVOL5**	20.44±2.92	186.33±27.38^**^
**EVOL6**	0.06±0.02	1.09±0.41^**^
**GPAT**	0.80±0.40	8.80±3.76^**^
**HMG-CoAR**	1.28±0.53	16.19±2.49^**^
**LDLR**	0.40±0.08	1.84±0.27^**^
**ABCA1-**	0.43±0.04	2.06±0.48^**^
**MTTP**	13.32±4.39	44.12±9.52^**^
**GLUT2**	15.46±3.30	4.36±2.10^**^
**PEPCK**	4.06±2.46	32.78±16.63^**^
**G6Pase**	0.92±0.12	0.97±0.09
**pyruvate kinase**	58.83±6.95	90.59±13.85^**^
**malic enzyme**	0.01±0.002	0.02±0.001^**^

Data are given as mean ± SD (n = 20, each genotype) in arbitrary units normalized to 18 S RNA contend. T-test C57Bl6 vs. alb-SREBP-1c: **p<0.01.

The hepatic expression level of genes was determined by RT-PCR (n = 20 each). The relative RNA amount shown in arbitrary units was calculated and plotted ± S.D. Students t-test was performed to determine significance (C57Bl6 vs. alb-SREBP-1c mice: **p<0.01).

Compared to C57Bl6 in alb-SREBP-1c mice, gene expression levels of lipid metabolic enzymes as fatty acid synthase (FAS), ω9 stearoyl-CoA desaturase (SCD), ω5 fatty acid desaturase-1 (FADS1) or ω6 fatty acid desaturase-2 (FADS2) were increased ∼3 to ∼10-fold (p<0.01) (Table 1). The expression levels of elongation of long chain fatty acids (ELOVL) family members ELOVL5 and ELOVL6 were increased ∼9-fold to ∼18-fold (p<0.01) respectively. Mitochondrial glycerol-3-phosphate acyltransferase (GPAT) expression level was elevated ∼10 fold (p<0.01). The rate-limiting gene in cholesterol metabolism (3-hydroxy-3-methylglutaryl-Coenzyme A reductase (HMG-CoAR)) was increased ∼12-fold (p<0.01). Genes with functional roles in lipid transport (such as microsomal triglyceride transfer protein (MTTP), low density lipoprotein receptor (LDLR), or ATP-binding cassettesub-family A-1 (ABCA1)) showed a ∼4-fold (p<0.01) increase in gene expression levels. Interestingly, the glucose transporter-2 (GLUT2) was down regulated in alb-SREBP-1c mice to ∼30% (p<0.01) of the values of C57Bl6 mice. Moreover, central rate-limiting metabolic genes such as phosphoenolpyruvate carboxykinase (PEPCK) were increased by ∼8-fold (p<0.01), while liver-specific pyruvate kinase or malic enzyme was increased nearly 2-fold (p<0.01). Glucose-6-phosphatase (G6Pase) was not altered. Taken together, over-expression of the N-terminal active domain of SREBP-1c in mouse liver alters the regulation of SREBP-1c target genes involved in DNL.

### Hepatic over-expression of the transcriptionally active domain of human SRREBP-1c affects liver morohology, histology and the hepatic lipid profile

**Figure 2:**
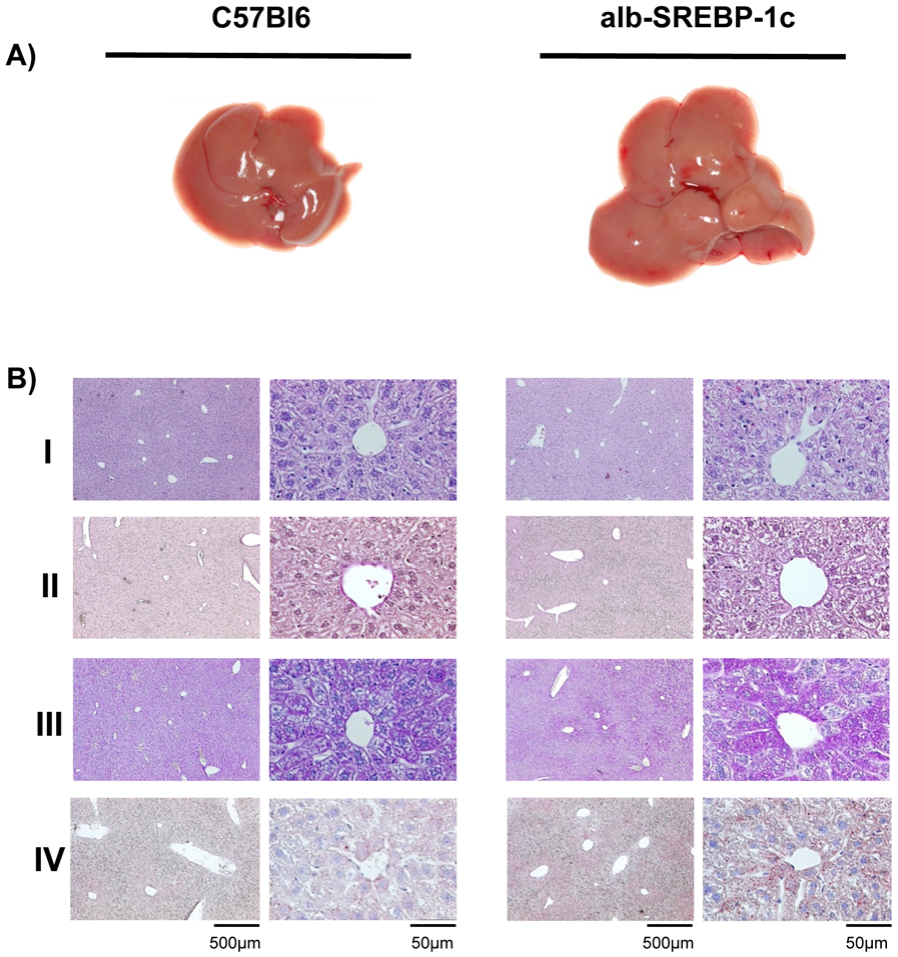
Macroscopic and histological comparison of livers from C57Bl6 and alb-SREBP-1c mice. Panel (A) shows fatty liver macroscopically of a C57Bl6 (left) or alb-SREBP-1c (right) mouse. (B) Liver tissue of the Lobus caudatus, Lobus sinister- and Lobus dexter lateralis were used for (I) standard hematoxylin and eosin staining. (II) PAS staining was performed to determine glycogen content. (III) The tissues were also used for cryofixation, and Oil-red-O staining was used for lipid visualization. (IV) Fibers and the extra cellular matrix were visualized to determine tissue integrity. The overview magnification is 1∶10, and details are shown in 1∶100 magnification.

For further analyses, C57Bl6 and alb-SREBP-1c mice were housed under standardized conditions and were fed a normal diet. Pathological examination of the mice at 24 weeks of age revealed slightly pale, enlarged livers for the alb-SREBP-1c mice (Figure 2A). Histological analyses of C57Bl6 liver tissues indicated morphologically intact parenchymatical structures with dense cytoplasm, clear nuclei, eosinophilic nuclei and basophile euchromatin. Oil-red-O stains showed several small lipid droplets but no signs of lipid accumulation. (Figure 2B). In alb-SREBP-1c mice, the general impression of the liver tissue was more dimorphous because the cellular structures revealed less dense cytoplasma with more vacuoles. Visible accumulation of lipid droplets, located mainly around the nucleus, was higher and centered around the portal vein, but not every cell was affected. In cells with the highest ectopic lipid accumulation, no signs of cytotoxicity in the form of degradation of the nuclear structures were obtained (Figure 2B). Further pathohistological alterations, such as dysmorphic cellular structures or infiltrations, were not detected. The expression of the human N-terminal of the transcriptionally active domain of SREBP-1c under control of the albumin promoter resulted in mild hepatic lipid accumulation under a standard diet.

A comparison of the liver percentile content of saturated fatty acids between C57Bl6 and alb-SREBP-1c mice (Table 2) indicated no difference for palmitic acid (C16∶0), but a 25% reduction for stearic acid (C18∶0) in alb-SREBP-1c mice (Table 2) was observed. The content of monounsaturated fatty acids (MUFA) was increased ∼3-fold (p<0.01) for palmitoleic acid (C16∶1) and increased 30% (p<0.01) for oleic acid (C18∶1). The content of polyunsaturated fatty acid (PUFA) as linoleic acid (C18∶2) or arachidonic acid (C20∶4) were reduced by ∼20% and ∼30% respectively (p<0.01), whereas α-linolenic acid (C18∶3) was increased by ∼30% (p<0.01) (Table 2).

**Table 2:** Fatty acid composition of liver.

genotype	C16∶0[%]	C16∶1[%]	C18∶0[%]	C18∶1[%]	C18∶2[%]	C18∶3[%]	C20∶4[%]
**C57Bl6**	29.98±0.62	1.20±0.07	15.83±0.57	13.98±0.95	20.38±0.65	0.35±0.05	18.13±1.06
**alb-SREBP-1c**	30.33±0.57	4.08±0.45^**^	12.10±1.45^**^	22.60±2.07^**^	16.28±2.26^**^	0.48±0.04^**^	12.68±1.43^**^

Fractional content of selected fatty acids. Data are mean ± SD (n = 20). T-test C57Bl6 vs. alb-SREBP-1c: **p<0.01.

The specific composition of total fatty acid was determined by GC analyses in liver tissues of C57Bl6 and alb-SREBP-1c transgenic animals (n = 20 for each). Students t-test was performed to determine significance (C57Bl6 vs. alb-SREBP-1c mice: **p<0.01).

Analyzing the data to determine the percentile change of TFA composition in the livers of alb-SREBP-1c mice compared to C57Bl6 mice mainly indicated a shift to MUFAs in the livers of the alb-SREBP-1c animals. These observations can be attributed to: fatty acids desaturation indices, which were significantly increased (∼3-fold (p<0.01) for C16∶0 to C16∶1 and ∼2fold (p<0.01) elevated for C18∶0 to C18∶1), a ∼30% (p<0.01) decreased elongation index and a ∼20% (p<0.01) elevated *de novo* lipid index in alb-SREBP-1c mice resulting from over-expression of human transcriptionally active N-terminal SREBP-1c domain in mouse liver (Table 3).

**Table 3:** Hepatic Δ9-desaturase, elongase and *de novo* lipidsynthese (DNL) Index.

genotype type	Δ9-Desaturase[C16∶1/C16∶0]	Δ9-Desaturase[C18∶1/C18∶0]	Elongase[C18∶0/C16∶0]	DNL[C16∶0/C18∶2]
**C57Bl6**	0.04±0.01	0.88±0.09	0.53±0.03	1.47±0.06
**alb-SREBP-1c**	0.13±0.02^**^	1.90±0.36^**^	0.39±0.05^**^	1.89±0.26^**^

Data are mean ± SD (n = 20). T-test C57Bl6 vs. alb-SREBP-1c: **p<0.01.

Fatty acid data were used to calculate desaturase index (C16∶0/C16∶1) and (C18∶0/C18∶1), elongation index (C18∶0/C16∶0) and *de novo lipid synthesis index* (C16∶0/C18∶2). Students t-test was performed to determine significance (C57Bl6 vs. alb-SREBP-1c mice: **p<0.01).

### Physiological examinations of increased DNL

**Figure 3:**
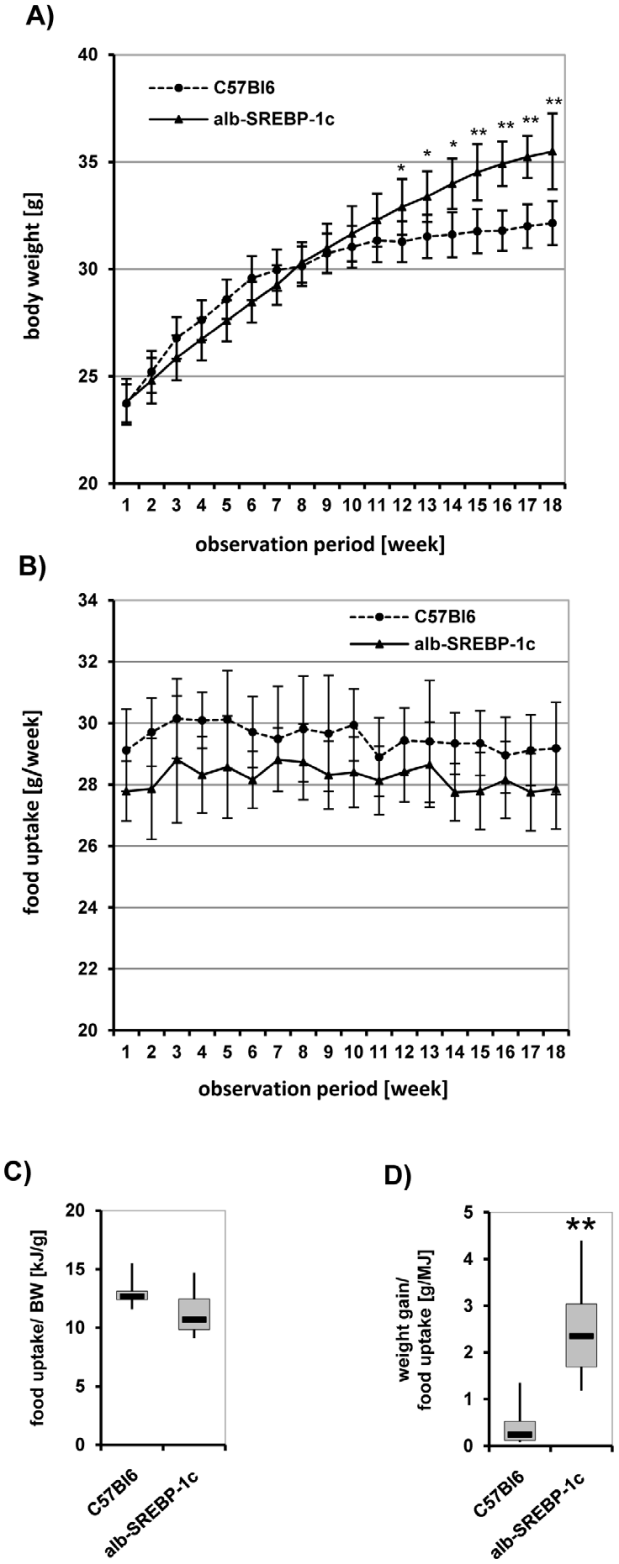
Phenotypical comparison of C57Bl6 and transgenic alb-SREBP-1c animals. Weight gain (A) and food intake (B) of male mice (n = 20 per genotype) were measured once a week starting at weaning and monitored for an observation period of 18 weeks. Food intake per body weight (C) and weight gain per food intake (D) were determined in each group of mice. Data are given as means including standard deviation (±S.D.). C57Bl6 vs. alb-SREBP-1c mice: **p<0.01.

To determine the systemic impact and especially the physiological consequences of increased DNL without further physiological stress, C57Bl6 and alb-SREBP-1c mice were housed under standardized conditions with unrestricted access to water and regular chow. No significant alterations in reaction to external stimuli and social behavior could be obtained for the alb-SREBP-1c mice. Viability, vitality, fertility and breeding behavior of the transgenic mice were comparable to the C57Bl6 mice. However, the survival rate of alb-SREBP-1c mice *postpartum* was reduced, with 17% of offspring dying within the first 3 days following delivery, compared to 6% of offspring of the C57BL6 controls. The remaining animals had a normal life expectancy without any special further treatment. Housing two mothers and their litter in one cage improved the number of surviving pubs independent of genotype. After weaning at 6 weeks of age, mice received a standard diet for a further 18 weeks until being sacrificed at the age of 24 weeks. At 6 weeks of age, the weight of alb-SREBP-1c mice was ∼10% (p<0.05) lower than C57Bl6 mice. At 15 weeks of age, the weight of C57Bl6 animals reached a plateau, but in alb-SREBP-1c mice, weight gain persisted until 24 weeks to approximately twice the value observed for C57Bl6 mice (p<0.01) (Figure 3A). Hyperphagia could be excluded because alb-SREBP-1c mice consumed 10% (p<0.05) less food than C57Bl6 animals (Figure 3B). Food intake by body weight was even slightly reduced in alb-SREBP-1c compared to C57Bl6 (Figure 3C), and weight gain per MJ food intake was more than 2-fold compared to C57Bl6 (Figure 3D).

**Figure 4:**
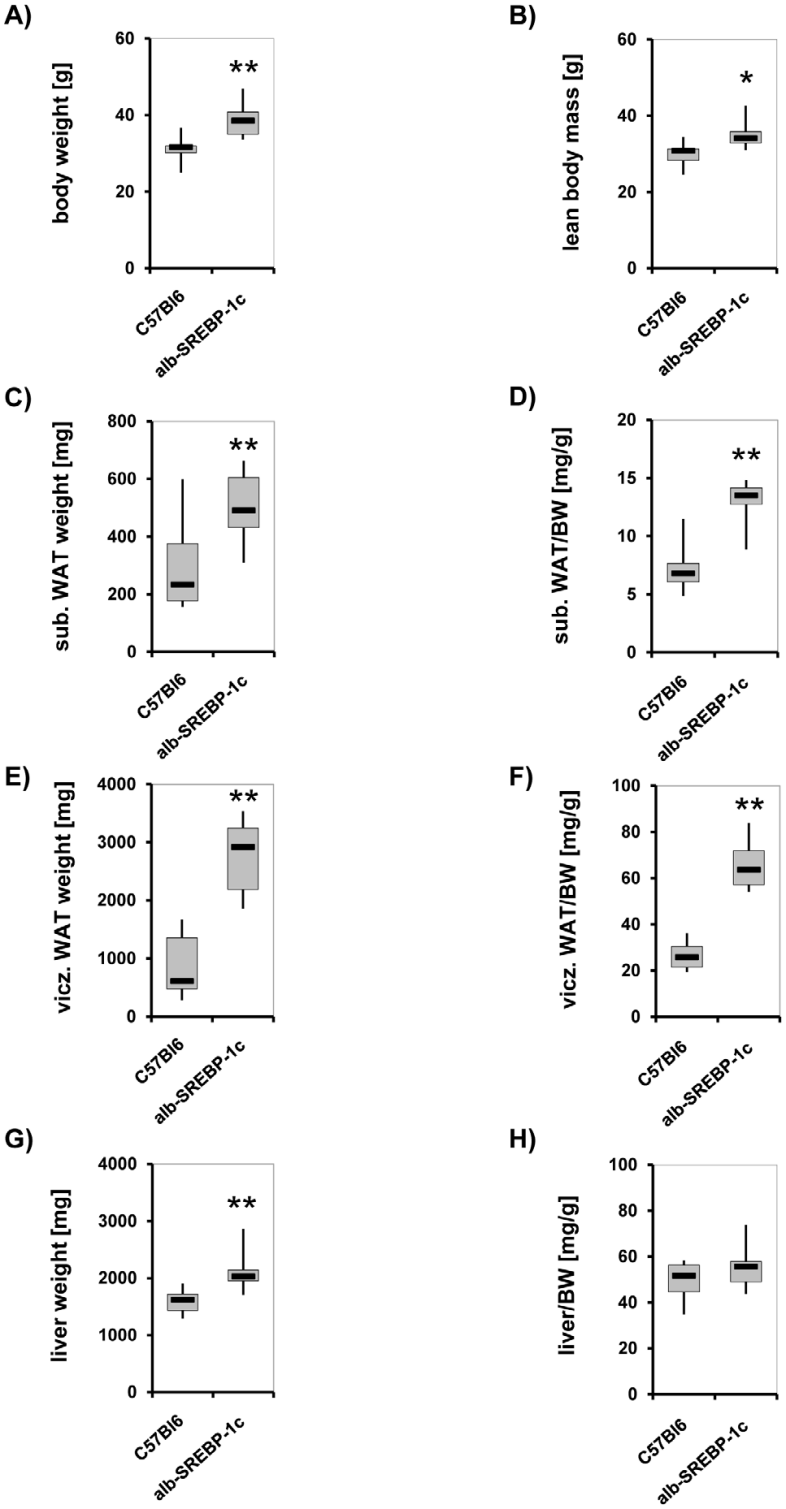
Comparison of body composition of C57Bl6 and transgenic alb-SREBP-1c animals. Body weight (A), lean body mass (B), subcutaneous adipose tissue (C), visceral adipose tissue (E) and liver weight were determined at scarification at 24 weeks of age of male mice (n = 20 per genotype) and are given directly (A, B, C, E, G) and in relation to body weight (BW) (D, F, H). C57Bl6 vs. alb-SREBP-1c mice: *p<0.05; **p<0.01.

At 24 weeks, total body weight of alb-SREBP-1c mice was approximately 20% (p<0.01) higher (Figure 4A). The lean body mass was increased, but not to the same extent (p<0.05) (Figure 4B). The total fat mass was increased more than 2-fold in alb-SREBP-1c animals compared with the C57Bl6 animals. The differentiation into subcutaneous and visceral fat mass indicated a total increase of visceral fat depots in alb-SREBP-1c; this increase occurred in correlation with body weight (Figure 4C, 4D). Visceral fat was increased more than 3-fold either directly (p<0.01) or in relation to body weight (p<0.01) in alb-SREBP-1c mice (Figure 4E, 4F). The mean liver weight had risen about 25% (p<0.01) at this time (Figure 4G), but the increase in liver weight relative to body weight was marginal (Figure 4H). Taken together, increased lean body mass and fatty liver were not the major sources of weight excess observed in the alb-SREBP-1c animals.

### Body composition is influenced by liver-specific overespression of human SREBP-1c

**Figure 5:**
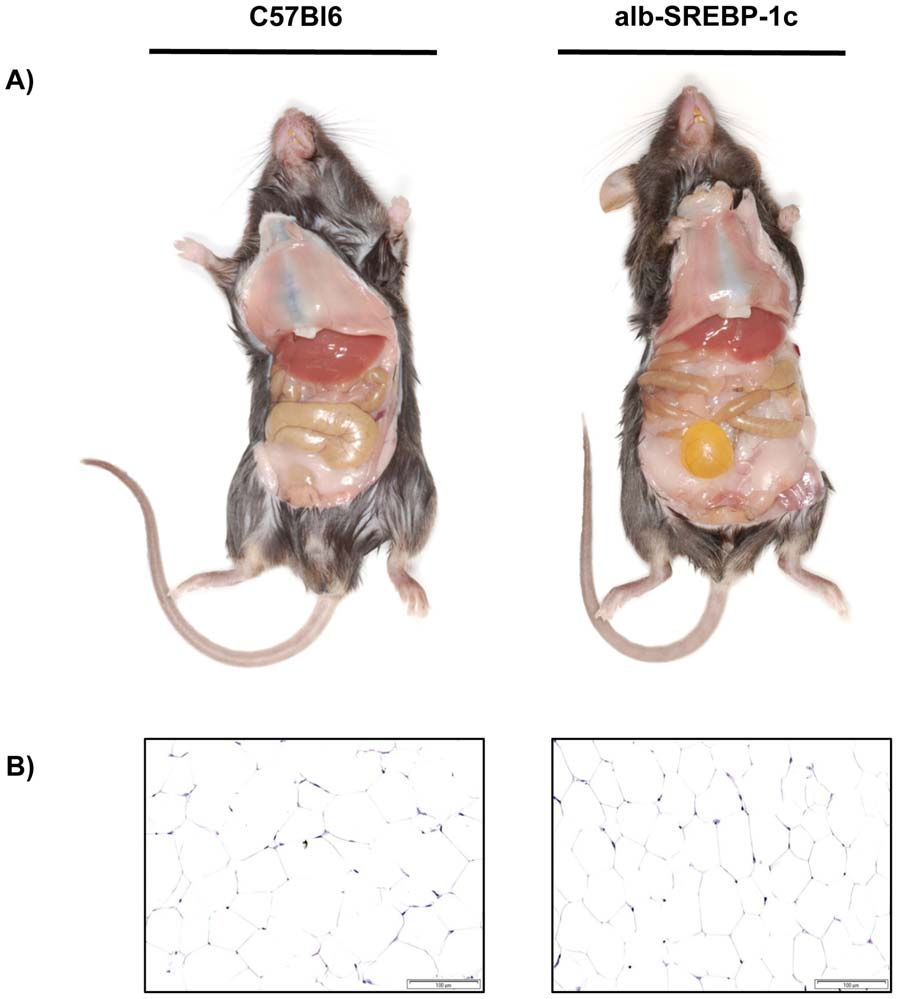
Macroscopic comparisons of C57Bl6 and transgenic alb-SREBP-1c animals. (A) First, sections of male mice at 24 weeks of age are shown. (B) Histology of visceral adipose tissue indicated hyperplasia but no signs of infiltration. All photographs were taken with the same magnification.

Macroscopic examination of alb-SREBP-1c mice at 24 weeks of age revealed pale, enlarged livers as expected from the fatty liver phenotype. Unexpectedly, the epididymal, gluteo-femoral and inguinal fat mass of alb-SREBP-1c was also greatly increased (Figure 5A). Histological examination of the adipose depots revealed simple hyperplasia but no hints for adipocyte hypertrophy or infiltrating macrophages (Figure 5B).

In adipose tissue, the detailed analyses of fatty acid composition did not indicate a significantly different lipid profile between C57Bl6 and alb-SREBP-1c animals (Table 4).

**Table 4:** Fatty acid composition of fat.

genotype	C16∶0[%]	C16∶1[%]	C18∶0[%]	C18∶1[%]	C18∶2[%]	C18∶3[%]	C20∶4[%]
**C57Bl6**	21.26±1.52	6.13±0.99	1.87±0.13	36.04±3.68	32.21±4.34	1.29±0.23	0.40±0.11
**alb-SREBP-1c**	23.60±1.52	6.42±0.84	2.05±0.24	34.37±1.32	31.17±1.45	1.28±0.21	0.33±0.05

Fractional content of selected fatty acids. Data are mean ± SD (n = 20).

The specific composition of total fatty acid was determined by GC analyses in adipose tissues of C57Bl6 and alb-SREBP-1c transgenic animals (n = 20, each). Students t-test was performed to determine significance (observed alterations did not reach significance limit p<0.05).

### Adiposity reduced the secreted cytokine pattern from adipocytes

**Figure 6:**
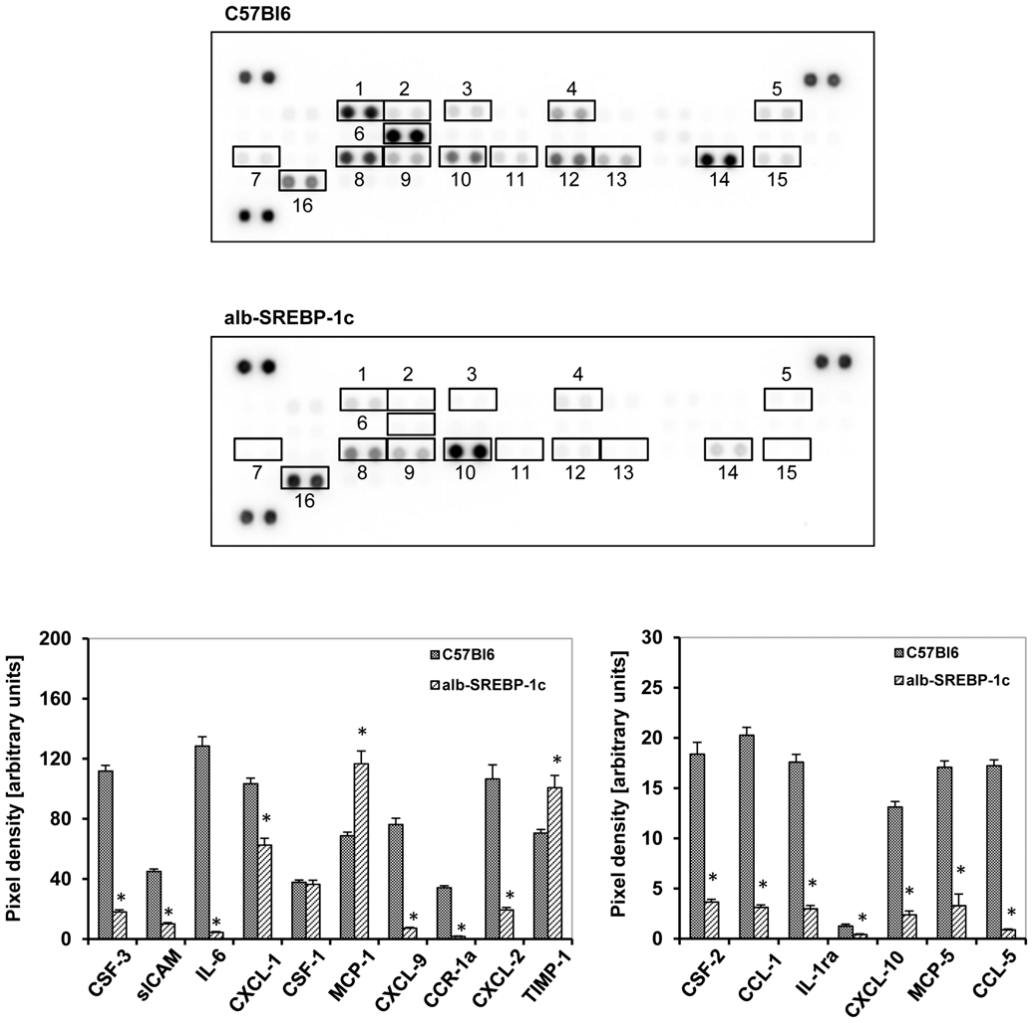
Cytokine profile secreted from isolated adipocytes of C57Bl6 and transgenic alb-SREBP-1c animals. The cytokine content in supernatant of cultured primary adipocytes was analyzed using the Proteome Profiler™; R&D Systems, (Abingdon, UK). Spot intensities were normalized to background and positive controls set to 100% intensity. Presented numbers on membranes mark targets as follows: (1) CSF-3; (2) CSF-2; (3) CCL-1; (4) sICAM -; (5) IL-1ra; (6) IL-6; (7) CXCL-10; (8) CXCL-1; (9) CSF-1; (10) MCP-1; (11) MCP-5; (12) CXCL-9; (13) CCR-1a; (14) CXCL-2; (15) CCL-5; (16) TIMP-1. Abundance of: CXCL-13, C5a, CCL-11, IFN-γ, IL1-α, IL1-ß, IL-2, IL-3, IL-4, IL-5, IL-7, IL-10, IL-13, IL-12-p70, IL-16, IL-17, IL-23, IL-27, CXCL-11, CCL-4, CXCL-12, CCL-17, TNF-α or TREM-1 was not detected. Data are given as means ± S.D. (n = 6, each) of normalized intensity. Significance was calculated by 2-way ANOVA. C57Bl6 vs. alb-SREBP-1c mice: *p<0.01.

There is an ongoing discussion that obesity and ectopic hepatic lipid accumulation is accompanied with increased inflammation [37]–[39]. To determine if the phenotype of obesity and hepatic lipid accumulation due to over-expression of human transcripitonally active N-terminal domain of human SREBP-1c is associated with increased secretion of inflammatory and proinflammatory parameters from adipose tissue, a set of 40 chemokines and cytokines was analyzed in the secretom of isolated adipocytes of control and transgenic animals (Figure 6). These arrays contain chemokines of the C-C motif ligands (CCL-) and receptors (CCR-),the C-X-C motif ligands (CXCL-) and receptors (CCR-), mast cell proteases (MCP-) or colony stimulating factor (CSF-) families, as well as intracellular adhesion molecules (ICAM), tissue inhibitor of metalloproteinase-1 (TIMP-1), complement components (C5a) or triggered receptor expressed in myeloid cells (TREM-1), in addition to various cytokines such as interleukines (IL-), interferone (INF-γ) and tumor necrosis facor (TNF-α). In C57Bl6 mice, an abundance of the chemokines CGF-3, CCL-1, sICAM, IL-1ra, IL-6, IL-12-p70, CXCL-10, CXCL-1, CSF-1, MCP-1, MCP-5, CXCL9, CCR-1a, CXCL2, TIMP-1 and CCL-5 was detected, but no proinflammatory cytokines could be determined. The pattern of cytokines detected in C57Bl6 and alb-SREBP-1c was comparable, and none of the cytokines expressed was specifically altered in abundance according to the genotype investigated. The analyses of the secretion profile of isolated adipocytes of alb-SREBP-1c mice indicated the same pattern, but in general, the quantity was lower except for MCP-1 and TIMP-1 (p<0.01).

### Clinical chemistry parameters in mouse serums

The surrogate parameters for liver function alanine amino-transferase (ALT) and aspartate amino-transferase (AST) were nearly doubled (p<0.01) in alb–SREBP–1c animals compared to C57Bl6 mice (Table 5). Cholesterol levels were unaltered, but triglycerides levels were increased∼2-fold (p<0.01) in alb–SREBP–1c mice. In accordance with increased fat mass, leptin levels were elevated ∼3-fold (p<0.01) as well. Serum-free fatty acids (FFA) as well as total fatty acid (TFA) in liver were increased ∼2-fold (p<0.01), whereas TFA in adipose tissue was not signifcantly altered (Table 4). Analyses of fatty acid composition in serum showed that C16∶1 and C18∶3 were slightly increased, but the alterations observed did not reach significance (p>0.05) (Table 6).

**Table 5:** Physiological parameters and serum lipid composition.

	C57Bl6	alb-SREBP-1c
**ALT [U/l]**	31.45±13.72	61.76±23.94^**^
**AST [U/l]**	30.22±19.55	71.00±23.49^**^
**cholesterol [mg/dl]**	106.08±16.71	106.53±20.83
**triglceride [mg/l]**	124.23±33.17	221.76±40.67^**^
**leptin [ng/ml]**	15.81±5.71	55.40±5.91^**^
**FFA serum [g/l]**	0.90±0.1	2.42±0.19^**^
**TFA liver [mg/g tissue]**	24.17±5.39	37.47±9.89^**^
**TFA fat [mg/g tissue]**	748.03±34.46	819.93±33.50

Data are mean ± SD (n = 20). T-test C57Bl6 vs. alb-SREBP-1c: **p<0.01.

Clinical parameters were measured in C57Bl6 and alb-SREBP-1c mice (n = 20, for each). Triglycerides cholesterol, total protein and liver enzymes (ALT, AST) were determined on a Hitachie 912 laboratory automat. Students t-test was performed to determine significance (C57Bl6 vs. alb-SREBP-1c mice: **p<0.01).

**Table 6:** Fatty acid composition of serum.

genotype	C16∶0[%]	C16∶1[%]	C18∶0[%]	C18∶1[%]	C18∶2[%]	C18∶3[%]	C20∶4[%]
**C57Bl6**	26.06±1.01	1.30±0.10	14.05±0.69	19.10±0.62	31.05±0.81	0.70±0.17	6.68±0.83
**alb-SREBP-1c**	25.73±0.95	1.73±0.55	12.50±1.28	19.90±0.86	30.45±1.27	1.05±0.11	7.98±0.65

Fractional content of selected fatty acids. Data are mean ± SD (n = 20).

The specific composition of free fatty acid was determined by GC analyses in serum of C57Bl6 and alb-SREBP-1c transgenic animals (n = 20, each). Students t-test was performed to determine significance (observed alterations did not reach significance limit p<0.05).

### Hepatic lipid accumulation does not alter serum cytokine pattern

**Figure 7:**
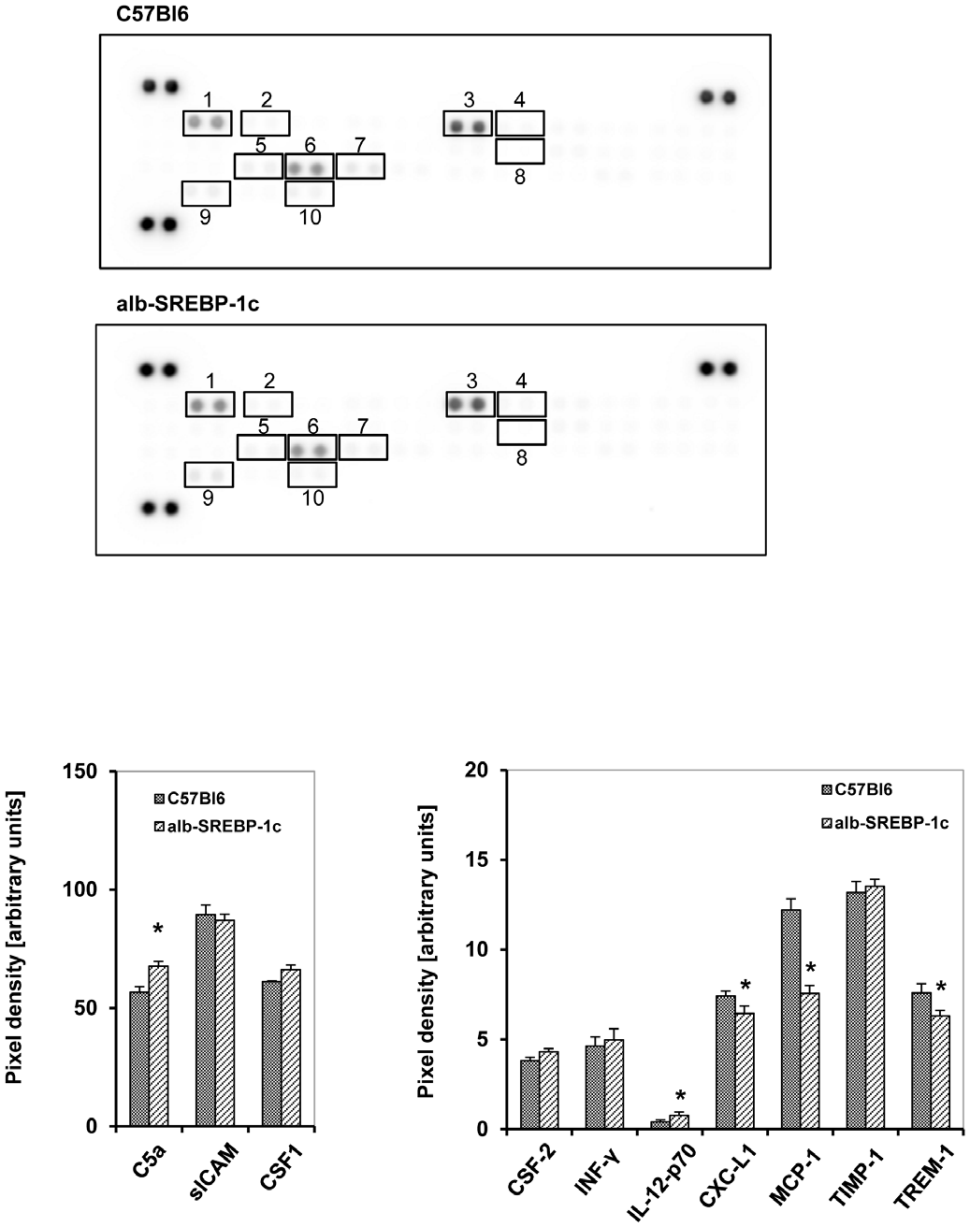
Cytokine profile in serum of C57Bl6 and transgenic alb-SREBP-1c animals. The cytokine content in serum was analyzed using the Proteome Profiler™; R&D Systems, (Abingdon, UK). Spot intensities were normalized to background and positive controls set to 100% intensity. Presented numbers on membranes mark targets as follows: (1) C5a; (2) CSF-3; (3) sICAM; (4) INF-γ; (5) IL-12-p70; (6) CXCL-1; (7) CSF-1; (8) MCP-1; (9) TIMP-1; (10) TREM-1. Abundance of: CXCL13, CSF-2, CCL-1, CCL-11, IL1-α, IL1-ß, IL-1ra, IL-2, IL-3, IL-4, IL-5, IL-6, IL-7, IL-10, IL-13, IL-16, IL-17, IL-23, IL-27, CXCL-10, CXCL-11, MCP-5, CXCL-9, CCR-1a, CCL-4, CCL-2, CCL5, CXCL-12, CCL-17 or TNF-α was not detected in serum. Data are given as means ± S.D. (n = 6, each) of normalized intensity. Significance was calculated by 2-way ANOVA. C57Bl6 vs. alb-SREBP-1c mice: *p<0.01.

The cytokine as well as chemokine pattern detected in serum of C57Bl6 and alb-SREBP-1c mice was comparable with no genotype-specific parameter (Figure 7). In C57Bl6, the analyses showed mainly an abundance of chemokines, i.e. C5a, CSF-3, sICAM, INF-γ, Il-12-p70, CXCL-1, CSF-1, MCP-1, TIMP-1 and TREM-1, but no proinflammatory cytokines could be determined (Figure 7). The pattern was comparable for alb-SREBP-1c mice and only C5a, and IL-12-p70 were elevated, whereas CXCL-1, MCP-1 and TREM-1 were less abundant in alb-SREBP-1c mice (p<0.01).

### Liver-specific overespression of human SREBP-1c influences parameters of systemic insulin sensitivity

Excess intracellular hepatic lipid accumulation can affect insulin sensitivity in human studies and mouse models [2], [3], [21]. In alb-SREBP-1c mice, blood glucose was nearly doubled (p<0.01) and insulin levels were particularly increased (∼4-fold (p<0.01)) compared to C57Bl6 mice (Table 7). The surrogate parameter for insulin resistance HOMA-IR was elevated ∼3-fold (p<0.01) and the surrogate parameter for insulin sensitivity QUICKI was reduced by 30% (p<0.01) in alb-SREBP 1c mice.

**Table 7:** Surrogate parameters for insulin resistance and insulin sensitivity.

	C57Bl6	alb-SREBP-1c
**blood glucose [mmol/l]**	7.82±0.99	11.01±1.39^**^
**insulin [ng/ml]**	0.95±0.53	3.56±1.11^**^
**HOMA-IR**	0.31±0.14	1,78±0.71^**^
**QUICKI**	0.47±0.04	0.34±0.02^**^

Data are mean ± SD (n = 20). T-test C57Bl6 vs. alb-SREBP-1c: **p<0.01.

Surrogate indices were calculated from fasting blood glucose and plasma insulin concentrations as follows: QUICKI = 1/[log(I0)+log(G0)], where I0 is fasting insulin (µU/ml) and G0 is fasting glucose (mg/dl); and HOMA-IR = (G0 * I0)/22.5, with glucose expressed as mmol/l and insulin expressed as µU/ml.). Data were calculated from all mice investigated. Students t-test was performed to determine significance (C57Bl6 vs. alb-SREBP-1c mice: **p<0.01).

## Discussion

Expression of the human N-terminal transcriptionally active domain of SREBP-1c under the control of the liver-specific albumin promoter in mice induces a mild fatty liver and an obesity phenotype. This is accompanied by altered body composition, altered lipid profile in liver and reduced insulin sensitivity, but not with increased inflammation.

The SREBP-1 isoforms are produced as inactive precursor proteins which are anchored in the endoplasmatic reticulum. The release of the transcriptionally active domain is tightly regulated by a step-wise proteolytic cascade [31], [33]. The direct use of the N-terminal domain of SREBP-1c makes the alb-SREBP-1c mouse model independent from this regulated proteolytic process release. Alb-SREBP-1c mice develop the observed phenotype under normocaloric conditions. This is the major difference in the model presented compared to the model of liver-specific over-expression of the human N-terminal transcriptionally active SREBP-1c domain under control of the PEPCK promoter [23]. In contrast to PEPCK promoter, the albumin promoter with the appropriate liver-specific enhancer we chose is strictly restricted to the liver from early development and continually remains highly active during the lifespan [40]–[43]. During embryogenesis, the PEPCK promoter is repressed due to high insulin levels in the fetal livers, and in adult animals, the PEPCK promoter is regulated by various stimuli in a feedback manner. Moreover, PEPCK expression is not exclusively restricted to the liver [44]. Furthermore, a special low carbohydrate/high protein diet is required to induce the fatty liver phenotype in PEPCK-SREBP-1c mice [23]. This might account for the differences observed, because alb-SREBP-1c and control mice can be directly compared on a diet of regular chow.

There were differences in the gene expression levels of SREBP-1c target genes, as no alteration in genes involved in cholesterol metabolism and a mild 2 to3-fold induction of genes involved in fatty acid metabolism were reported in PEPCK-SREBP-1c mice [23].

In general, the gene expression profiles in livers of alb-SREBP-1c mice are comparable to the gene regulatory action of SREBP-1c in various cell models [22], [23]. LDLR or HMG-CoAR was up-regulated in livers of the alb-SREBP-1c animals and in cell models but unaltered in the livers of PEPCK-SREBP-1c mice [23]. Key enzymes of lipid metabolism, such as FAS, ω9-, ω5- and ω6-desaturases SCD, FADS1 or FADS2, or the elongases ELOVL5 and ELOVL6, indicated increased mRNA levels in alb-SREBP-1c mice. A positive correlation between ω9-desaturase SCD mRNA level and the degree of lipid accumulation can be drawn in alb-SREBP-1c mice, which is in contrast to human studies, where a negative correlation between SCD expression and the degree of hepatic lipid accumulation has been shown [45]. This might be directly due to SREBP-1c over-expression in alb-SREBP-1c mice, and not the hepatic lipid accumulation induced.

The key enzyme GPAT, crucial in cellular TG synthesis, is also known to be regulated by SREBP-1c. Depletion of GPAT has been shown to improve hepatic steatosis and associated insulin resistance [46].

Genes involved in transport processes concerning serum lipid homeostasis are increased in alb-SREBP-1c mice. Especially MTTP is necessary for the formation of very low density lipoprotein (VLDL) particles and to shuttle TG out of the liver [47]. As in MTTP deficient mice, the TG levels are low as export does not function they show hepatic steatosis but lack insulin resistance or inflammation [48]–[50]. The increased expression of MTTP mRNA in alb-SREBP-1c mice might be an indicator of increased TG efflux in the livers of alb-SREBP-1c mice.

In summary, hepatic gene expression indicated a substantial increase in expression of lipogenic enzymes; this was expected from the model of SREBP-1c over-expression which resulted in putatively increased DNL, thereby facilitating TG production and accumulation in hepatocytes.

In alb-SREBP-1c mice, plasma cholesterol is unaltered, but the serum TG levels are increased. The desaturation of fatty acids is significantly increased, whereas the elongation index shows a decrease and *de novo* lipid index is slightly increased. This indicates an increase of desaturated fatty acids that are able to be stored in the liver.

The onset of hepatic steatosis is still not clear, and a common thought is that accumulation of lipids is initiated by an increased release of fatty acids from the adipose tissue into the plasma. Although the composition of fatty acids in liver in the mouse model is different from the C57Bl6 control, the fatty acid pattern of serum or adipose tissue indicates only marginal differences. In view of our hypothesis, this could indicate that systemic transport systems are not the primary source of hepatic lipids, but rather the DNL in liver results in increased production.

Although the alb-SREBP-1c mice develop a fatty liver, this phenotype is rather mild; the more striking observation is the vast increase of visceral adipose tissue. This is the marked difference in the phenotype of the mice. The PEPCK-SREBP-1c mice show a mild fatty liver with slightly increased liver weight and increased liver TG content as our model does, but adipose tissue is rather reduced so they do not develop the obesity phenotype [23]. This increased visceral obesity in alb-SREBP-1c is due to adipose tissue hyperplasia and not associated with an altered fatty acid composition in adipose tissue or histological indicators of increased inflammation.

Obesity and ectopic hepatic lipid accumulation are thought to be accompanied by increased inflammation and circulating inflammatory parameters [37], [38]. There is still debate on whether inflammation is a cause or a consequence of obesity and hepatic lipid accumulation [39]. It is interesting to note that despite hepatic lipid accumulation and massive visceral obesity in alb-SREBP-1c mice, no gross alteration in the cytokine profile in serum can be observed, and the cytokine profile secreted by adipocytes indicates even a reduced abundance for most cytokines compared to C57Bl6 mice. The missing indication of inflammation could be a hint that at the current stage of lipid accumulation in the liver, there has not yet been a conversion to a pathological condition generally or in terms of inflammatory reactions. Also, locally restricted inflammation cannot be excluded, as well as alterations in inflammatory parameters below the sensitivity of the method chosen.

Only two chemokines, i.e. MCP-1 and Timp-1, are overrepresented in alb-SREBP-1c compared to C57Bl6 mice. MCP-1 has been shown to be secreted by adipose tissue in positive correlation with adiposity and features of the metabolic syndrome in humans and mice [51]. Timp-1 is secreted by adipocytes and involved in cellular matrix remodeling, thus playing a role in the plasticity of adipocytes [52]. Interestingly, adipogenesis blunts Timp-1 production [53]. So the data received from our model indicate that if inflammation is involved in the pathogenesis of either fatty liver or visceral adipositas, it is a secondary effect and not an initial event.

To determine, whether the fatty liver with visceral obesity in alb-SREBP-1c mice is associated with insulin resistance, we employed surrogate indices of insulin resistance HOMA-IR as well as surrogate indices of sensitivity QUICKI. Surrogate indices of insulin sensitivity and resistance have been successfully adapted to mice using the surrogate parameters developed for humans [54]–[60]. These analyses show a mild insulin resistance. Clinical studies have shown that the intracellular amount of lipid in liver is associated with insulin resistance [45], [61], [62]. The insulin resistance in our mice model is still weak and does not seem to affect the functionality of ß-cells yet. These observations stress the hypothesis that the system can compensate a certain genetic predisposition and increased lipid production.

In summary, our investigations imply that lipid accumulation in liver can occur due to the triggering of lipid metabolism by direct over-expression of a central player in lipid metabolism in liver, i.e. SREBP-1c. Furthermore, our observations stress the hypothesis that fatty liver is an initial parameter in the development of adipositas independent of environmental factors. The altered regulation of genes essential in hepatic lipid synthesis, a shift in the liver fatty acid pattern to more storage-capable saturated FA, and the moderate hepatic lipid accumulation in combination with a vast increase of adipose tissue observed in this model all favor the hypothesis that the liver has to be a more active player in metabolic diseases and the initialization of adipositas. In the mouse models presented, it is not that obesity is the initial key that induces the fatty liver, but rather *vice versa* that the initial failure in hepatic lipid metabolism accounts for the complete shift towards a metabolic disorder and adipositas without physiological challenge.

The question as to why alb-SREBP-1c mice develop such a vast amount of adipose tissue remains to be answered, but it seems to be reasonable that two mechanisms play a major role: i.e. either increased dietary lipids or increased DNL. In our mice model, over nutrition is not the main issue. Naturally, adipose metabolism was of prime importance for animals to survive states of starvation due to its efficient energy use of available food; therefore, induction of DNL due to SREBP-1c might be of importance. DNL plays an important role in this setting of positive fat balance. The best known physiological model of massively increased DNL is in pre-hibernating animals. Here, DNL is increased, and lipids produced were stored in adipose tissue. However, usually this is accompanied by increased food intake. How the hormonal regulation and metabolic control of this specific physiological condition is regulated is not known in excessive detail, but altered gene regulation on various levels is at least involved in the process [63]. If and how the sole over-expression of SREBP-1c in liver is necessary or sufficient to enable the system to obtain more storable energy of the food consumed, remains to be elucidated. One can speculate that according to the normal food consumption of alb-SREBP-1c mice, an increase of caloric intake can only be achieved by specific high fat diets. In this case, one could speculate that the phenotype of alb-SREBP-1c mice will worsen as soon as adipose tissue can no longer compensate and store excess lipids. But according to other morbid obese mouse models, the metabolism might reach a point of exhaustion up to cachexia. In this context, it would also be interesting if the model turns out to be useful to distinguish the impact of different dietary compounds such as high fat diets or high carbohydrate diets; but the effects of special physiological challenges and specific diets on these mechanisms remain to be established.

In conclusion, based on the description of the alb-SREBP-1c mouse model here, this mouse model allows the elucidation of the systemic impact of this central regulator of lipid metabolism *in vivo*. Furthermore, this model allows the opportunity to investigate the pathophysiology of primary lipid accumulation in liver as well as the long term effects of chronic fatty liver independent of physiological stress.

## Materials and Methods

### Generation of transgenic alb-SREBP-1c-NT mice

A vector, based on pBlueskript II KS (Stratagene,), was generated by inserting 2 kb of albumin enhancer sequence corresponding to the NheI/BamHI enhancer fragment [64] and the mouse albumin promoter (−308 to +8) containing all relevant transacting elements [40] into the BamHI site. All necessary inserts were generated by PCR from mouse genomic DNA. Subsequently, a polyA cassette was inserted via EcoRV/KpnI sites. Into this construct, the N-terminal transcriptionally active domain (SREBP-1c-NT; aa 2–436) including a 5′-HA-tag (YPYDVPDYA, the epitope of influenca hemagglutinin (HA)) was inserted using the reconstituted EcoRV/BamHI sites. The SREBP-1c expression cassette was released by BssHI restriction for microinjection into male pronuclei of zygotes derived from C57Bl6 mice.

### Animals, phenotypic and metabolic indices

Male C57Bl6 and alb-SREBP-1c mice (n = 20, each) were bred and maintained in colonies of four animals in our animal facility (12 h light/dark cycle; 22°C±1°C, 50%±5% humidity). They were fed *ad libitum* with standard laboratory chow (13.7 mJ/kg: 53% carbohydrate, 36% protein, 11% fat (Ssniff, Soest, Germany)) and had free access to water. At the age of 24 weeks, mice were sacrificed by CO_2_ asphyxiation. Mice of each genotype were also investigated in the collaborating Institute for Diabetes Research to verify physiological and histological parameters in an independent habitat. The Animal Care Committees of the University Duesseldorf and Hamburg approved animal care and procedure (Approval#50.05-240-35/06 and #93/08).

### Genotyping

Genomic DNA was extracted from ear biopsies taken at weaning with a tissue kit (Qiagen, Hilden, Germany) according to manufacture's instructions. Routinely, 50 ng DNA was used for PCR with SREBP-1c-NT (5′-TAGGCCAGGGAACTGACTG-3′) and albumin promoter (5′-ATGCGAGGTAAGTAT-3′) specific primers.

### Preparation of cell extracts and Western Blot analyses

To detect the expressed HA-SREBP-1c at protein level, nuclear extracts were prepared according to [65] from 10 mg of snap-frozen tissue samples. An antibody against HA peptide conjugated with peroxidase (clone 3F10, 1∶5000, Roche, Mannheim, Germany) was used for western blot detection. For normalizing, blots were probed with α-tubulin antibody (Santa Cruz). Visualization was performed with ECL™ plus Western Blotting detection reagents using a Versadoc instrument (BioRad).

### RNA extraction and real time (RT)-PCR analyses

For expression analyses, 10 mg liver tissue per individual animal was snap frozen in liquid nitrogen immediately after liver resection. Biopsies were lysed with Qiazol (Qiagen, Hilden, Germany). Total RNA extraction, Real time (RT) PCR analyses (Assay on Demand™, 2× Universal PCR Mastermix, ABI Prism 7000 Sequence Detection System Applied Biosystems, Darmstadt, Germany) and specific detection of endogenous mouse SREBP-1a and SREBP-1c isoforms were performed with mSREBP-1aFP 5′GAGGCGGCTCTGGAACAGA3′ or mSREBP-1cFP 5′GGAGCCATGGATTGCACATT3′, common reverse primer mSREBP-1RP 5′CACTGTCTTGGTTGTTGATGAGCTG3′, and specific probe SREBP-1(a/c) [5′]6-FAM TATCAACAACCAAGACAGTGACTTCCCTGGC [3′]TAMRA as previously reported [38]. The expression of the HA-SREBP-1c transgene in liver was confirmed with human specific primers (5′ATGTGGCAGGAGGTGGAGAC3′), a primer derived from the HA-tag sequence (5′TACGACGTCCCAGACTACG3′) and the identical probe. Data were normalised to 18 S RNA content.

### Clinical chemistry parameters

Blood parameters were measured at 24 weeks of age (n = 20). Blood glucose was measured with Freestyle™, and triglycerides, cholesterol, total protein and liver enzymes (ALT, AST) were determined on a Hitachie 912 laboratory automat (Roche, Mannheim, Germany). Liver biopsies were minced in NaCl solution (0.9% (w/v), pH 7.5) and homogenized using an ultraturax. To determine fatty acid content, serum or homogenate from liver biopsies minced in NaCl solution (0.9% (w/v), pH 7.5) were pelleted by centrifugation (800 g for 5 min at 4°C), and the pellet was subjected to fatty acid quantification. For gas chromatography (GC) analyses, sample preparation and resolution was performed as described [65]. Serum insulin levels (mU/l) were measured in triplicate by ELISA according to the manufacture's recommendation (Mercodia, Uppsala, Sweden).

### Histology

Tissues of the Lobus caudatus, Lobus sinister- and Lobus dexter lateralis were fixed in 4% paraformaldehyd/PBS and embedded in paraffin with automated standard histological procedures (Excelsior™, Thermo Shandon GmbH, Frankfurt, Germany). Standard hematoxylin and eosin (HE) staining was performed on 3 µm deparaffinized sections. Glycogen storage was monitored with PAS staining (Merck, Darmstadt, Germany). Fibers and the extra cellular matrix were visualized using the “van Gierson kit” (Merk, Darmstadt, Germany). For oil red O staining, cryoprotected liver was frozen under liquid-nitrogen-cooled isopentane and stored in liquid nitrogen until proceeding. Tissue sections were stained with oil red O solution, rinsed excessively with water, and retained dye was eluted by 40% isopropanol followed by H_2_O.

### Determination of the cytokine profile in serum and adipocytes

For parallel detection of various cytokines, serum or concentrated supernatant of adipocytes of C57Bl6 and alb-SREBP-1c mice were hybridized with array membranes according to the protocol supplied by the manufacturer (Proteome Profiler™; R&D Systems, Abingdon, UK). Serum samples were proceeded directly. For analyses of cytokine profiles of adipocytes derived from the visceral compartment, conditioned medium was prepared from freshly isolated adipose tissue according to [66]. The fractionated supernatants were concentrated by using filtration devices with a cutoff of 3 kDa (Amicon Ultra, Millipore, Bad Schwalbach, Germany) to a final volume of 200 µl culture medium/g of adipose tissue starting material. Six replicates per genotype were analyzed pairwise (blot 1: C57Bl6 vs blot 2:alb-SREBP-1c) and handled in parallel troughout the entire procedure. Exposure of each pair of arrays was also performed in parallel. and data were collected and processed as one file (Versadoc instrument (BioRad)). For normalization, local background hybridization signals were substracted from the spot intensities, and each spot was normalized to the internal hybridization positive controls. Normalised data were analysed pairwise for genotype-specific differences as processed, and results per pair were used to determine the mean differences in cytokine abundance.

### Statistical analysis

Values are presented as means ± SD. Statistical analyses were performed via either the Student T-test or by 2-way ANOVA using Prism 4.03 (GraphPad Software Inc., San Diego) as indicated.
